# Detection of Single Cell Contamination of *Salmonella* in Foods by SALX System and NIHSJ-01 and Estimation of LOD_95_

**DOI:** 10.14252/foodsafetyfscj.D-25-00008

**Published:** 2025-06-27

**Authors:** Hideaki Matsuoka, Takatoshi Moriyama, Natsuko Koshimizu, Norimasa Takatani, Tomonori Yoshida, Yoshiomi Shimabara, Tomoe Hirai, Kazuhide Nakajima, Shizunobu Igimi, Mikako Saito

**Affiliations:** 1 Department of Biotechnology and Life Science, Tokyo University of Agriculture and Technology, 2-24-16, Naka-cho, Koganei, Tokyo 184-8588, Japan; 2 Neogen Japan K.K., 3-3-3 Minatomirai, Nishi-ku, Yokohama, Kanagawa 220-0012, Japan; 3 Japan Research & Development Division, Nichirei Corporation, 9, Shinminato, Mihama-ku, Chiba 261-0002, Japan; 4 Tokyo Meat Safety Inspection Center Co.,Ltd, 5-1-30 Konan, Minato-ku, Tokyo 108-0075, Japan; 5 Food Safety Research Center, Tokyo University of Agriculture, 1-1-1 Sakuragaoka, Setagaya-ku,Tokyo 156-8502, Japan

**Keywords:** LOD_95_, NIHSJ-01, *Salmonella*, SALX System, single cell contamination, 10×10 sorting plate.

## Abstract

A novel idea of statistical analytical procedure for the level of detection (LOD) was demonstrated by its successful application to qualitative test methods for *Salmonella*, SALX System and NIHSJ-01. *Salmonella* cells of a hydrogen sulfide-producing strain FSD287 and a non-hydrogen sulfide-producing strain FSD347 were added to beef and shrimp food samples using a cell sorter to achieve bacterial cell concentration of 1, 5, and 10 cfu/25g-test portion (tp). The colony forming probability (CFP) of the added cells was estimated by means of 10×10 sorting plates. All of test portions containing FSD287 or FSD347 were decided to be positive by SALX System. NIHSJ-01 using CHROMagar® *Salmonella* (CHS) decided test portions of each of both strains to be positive, while NIHSJ-01 using desoxycholate hydrogen sulfide lactose (DHL) agar decided selectively only FSD287-test portions to be positive. All blank test portions were negative. To evaluate the level of detection at 95% probability (LOD_95_), in addition to test results, we introduced virtual results of blank conditions approaching zero. As a result, LOD_95_ for every case was estimated to be lower than 0.326 cfu/tp indicating that both methods were able to detect 1 cfu/tp at higher than 95% probability. Therefore, our protocol for statistical analysis for LOD was feasible for the verification of the test methods that meet the requirement of detecting small number (minimum 1 cfu/25g-tp) of target micro-organisms in food test portions.

## 1. Introduction

In Japan, the Food Sanitation Law and the Order on Milk and Milk products set acceptable limits of microbial contamination in foods. Microbial targets are, for instance, for coliforms, enterococci, *Pseudomonas aeruginosa*, *Salmonella* spp, *Vibrio parahaemolyticus*, *Listeria monocytogenes* and *Staphylococcus aureus* as microbial criteria for various foods. Qualitative tests are required to detect small number (minimum 1 cfu/test portion (tp)) of target micro-organisms in food test portions. Therefore, qualitative tests with applicable performance are required, and standard contaminated foods are needed to verify that performance.

Regarding standard contaminated foods, their preparation protocol is fundamentally to repeat 10-fold dilutions starting from a high concentration (e.g. 10^8^ cells/mL) to <10 cells/mL. The actual concentration of the final dilution is confirmed by colony counting method. If it is n cells/mL, the final dilution is diluted to 1/n to obtain a cell suspension of 1 cell/mL. Then it is further diluted to 1/25 to prepare a 25 g test portion containing 1 cell. Even if this protocol is carefully performed, it is still uncertain whether every test portion really contains only one target cell.

On the other hand, a cryopreserved material of definite small number (3~5) of viable bacteria was developed by using a cell sorter and freeze-drying processes^1^^,^^2^^)^. Each water drop dispensed by the cell sorter contains one viable bacterium, which can be stored for the use at any time. Such a material is commercialized under the name of BioBall®. Recently, International Organization for Standardization (ISO) 16140-3: 2021 has introduced a protocol using food test portions containing definite small number of target cells in the estimation of the level of detection at 50% probability (eLOD_50_)^3^^)^. It is likely that this protocol was introduced in light of the widespread availability of products such as BioBall.

However, the number of species and strains of bacteria available as BioBall is limited. Moreover, it seems to be still difficult to guarantee the cryopreservation stability of every one bacterial cell. Then we investigated a method of using a material that does not require long-term storage. Using a cell sorting equipment, live bacterial cells are added directly to food test portions on-site and are ready for use within a day, where the viability of the individual cells being sorted is crucial. Therefore, based on the flow cytogram, a cell fraction with high viability was selected for each bacterial species. It was demonstrated that single viable cell with colony forming unit could be sorted at higher than 95% probability for all of 16 species of randomly selected bacteria^4^^,^^5^^)^.

According to ISO 16140-2: 2016, and ISO 16140-3: 2023, the detection sensitivity of qualitative test methods is evaluated by the level of detection at 50% probability (LOD_50_). Statistical analysis of LOD is based on the Poison distribution^6^^,^^7^^)^ and a downloadable program for the analysis is available from ISO website^8^^)^. In this analysis, however, at the lowest cell concentration, at least one negative result is necessary to estimate LOD_50_ value. Formerly, we obtained the data that all of test portions containing one cell were positive by a qualitative test method, suggesting that the test method was highly sensitive (unpublished data). Nevertheless, the LOD_50_ of the test method could not be determined.

Through discussions with statistics experts in ISO activities, we have come up with a new idea of reasonable approximation method, which has resolved the previous issues and enabled us to evaluate the LOD of a high-performance test method capable of detecting 1 cfu/tp. Here, we set LOD_95_<1.0 cfu/tp as its criteria, since this value was thought to be more rational than LOD_50_<1.0 cfu/tp. The objective of this study is to demonstrate this new idea of statistical analytical procedure and its successful application to existing test methods for *Salmonella*, SALX System^9^^,^^10^^)^ and NIHSJ-01^11^^)^.

## 2. Materials and Methods

### 2.1 Microorganisms

A hydrogen sulfide producing (H_2_S(+)) strain, *Salmonella* Typhimurium FSD 287and a hydrogen sulfide non-producing (H_2_S(-)) strain, *Salmonella* Westhampton FSD 347were provided by Neogen (Lansing, MI, USA) (formerly 3M Food Safety, St. Paul, MN, USA) and cultured on standard methods agar(Merck, casein peptone 0.5%, yeast extract 0.25%, dextrose 0.1%, agar 1.5%, pH 7.0±0.2) at 37°C.

### 2.2 Cell Sorting

Each strain was precultured in tryptic soy broth (TSB) (Becton Dickinson Co. (BD), Cockeysville, MD, USA), and its cell suspension was plated on tryptic soy agar (TSA) (BD). After culture at 37°C for 18 h, the resulting colonies were picked up and suspended in a phosphate buffer solution (PBS) (0.1 M, pH 7.0). The cell concentration was roughly estimated from turbidity and then a dilution series was made for checking with a hemocytometer. Based on the results, the cell suspension adjusted at 10^8^ cells/mL was prepared. An aliquot of 1 mL from this cell suspension was transferred to a microtube, then 6-carboxyfluorescein diacetate (CFDA) (Sigma, St. Louis, MO, USA) was added to the microtube at 150 µg/mL. After the reaction with CFDA at 30°C for 30 min, the cell suspension was diluted 100-fold with PBS and then transferred into a 5 mL polystyrene round-bottom tube (BD Falcon, 352235) through a strainer cap attached to the tube. The mesh size of the strainer was 35 µm. The CFDA-stained cells were applied to a fluorescence-activated cell sorter (FACSAria II; BD).

The cell sorter enables setting the number of droplets (containing single cell/droplet) to drop on the food sample. The fluorescent intensity of each droplet is measured during dropping. If a droplet has the fluorescence intensity equivalent to one cell, an electric field is applied at the moment the droplet passes between the electrodes during its descent to change its direction of fall. The timing is critical and requires high precision. Practically, however, 100% certainty cannot be guaranteed and it is crucial to check the actual probability in the case that the number of cells to be collected is extremely small. Therefore, we have applied the sequential sorting plan described below to check this probability.

### 2.3 Sequential Sorting Plan

**Fig. 1.**
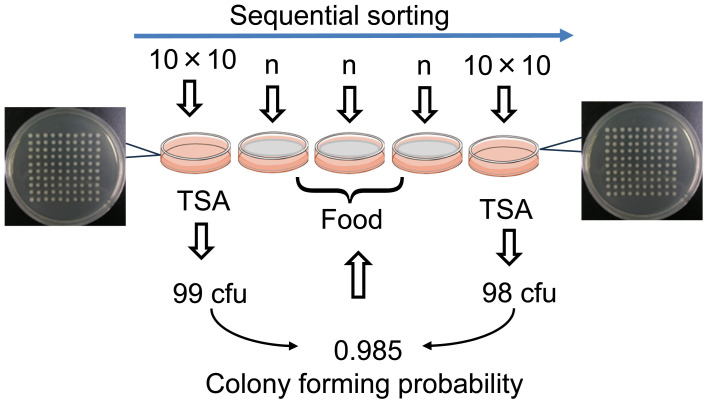
Sequential sorting plan for estimating CFP. Sorting on TSA: 100 cells in a 10×10 grid pattern. Sorting on food test portion: n cells without position control. CFP: number of colonies grown on a TSA plate/100 sorted viable cells. Estimated number of cells with colony forming unit (cfu) sorted on a food test portion: n×CFP. A cell sorter is a device that selects only droplets that contain a single live bacterium (CFDA positive) from a series of droplets that are continuously dropped at high speed. However, there are rare cases where the bacteria in the selected droplets have low activity of colony formation or are dead. Therefore, to ensure accuracy, it is important to estimate the relative number of viable cells maintaining colony forming activity. This probability value was determined from the ratio of the number of colonies per 100 (10×10) sorted cells. Thus, estimated value was denoted CFP, a probability of colony forming ability of each sorted cell.

Sequential sorting plan (Fig. 1) was conducted to estimate the CFP of each single cell sorted on food test portions. The sorting of 10×10 cells was conducted on TSA before and after the cell sorting on three food test portions. When the 10×10 cell sorting results are 99 cfu and 98 cfu, for instance, CFP is estimated as 0.985 (the mean of 2 values, 0.99 and 0.98). When n cells are sorted on a food test potion, expected cfu of each test portion is 0.985×n. If the probability is lower than 0.95, those test portions are excluded.

### 2.4 Food Samples

Fresh raw beef was collected from a cow in a slaughter house and cut into small blocks. One meat block was weighed and its 25 g test portion was cut from the block. Then the test portion was homogenized and transferred into a glass petri dish with a diameter of 90 mm. In the petri dish, a powder paper was laid beforehand for the convenience of smooth transfer of the meat homogenate into a stomacher bag.

Twenty four test portions were prepared at once and used for 4 conditions of cell quantity (0, 1, 5, 10 cfu/tp) × 3 repeats × 2 methods for *S.* Typhimurium FSD 287. Using another set of 24 test portions, the same test was conducted for *S.* Westhampton FSD 347.

Freshly frozen and stored shrimp was thawed before use, and its 25 g test portion was collected and homogenized. Successive experimental protocol was same as that for beef.

The contamination of non-specific bacteria in food test portions were checked by the colony count method using TSA medium (35°C, 48 h).

### 2.5 SALX System

SALX System is produced by Neogen and certified as a validated test kit, AOAC PTM 061301 and AOAC OMA 2014.01. The test protocol was provided by the producer as roughly described below. SALX System includes the following products:

(a) Neogen® Petrifilm® *Salmonella* Express Plate (hereafter SALX Plate).

(b) Neogen® Petrifilm® *Salmonella* Express Confirmation Disk (hereafter SALX Disk).

(c) Neogen® Petrifilm® *Salmonella* Enrichment Base (hereafter SEB).

(d) Neogen® Petrifilm® *Salmonella* Enrichment Supplement (hereafter SESUP001).

(e) Rappaport-Vassiliadis R10 Broth (hereafter RV-R10)

A 25 g test portion was combined with 225 mL enrichment medium (SEB and SESUP001) in a stomacher bag and homogenized thoroughly for 2 min and then incubated at 41.5±1°C for 1824 h. An aliquot of 0.1 mL of the primary enrichment was transferred into 10.0 mL RV-R10 and incubated at 41.5±1°C for 824 h. Then, an aliquot of 10 μL of the culture medium was sampled with a sterile smooth loop with 3 mm in diameter and streaked once onto the gel surface of a SALX plate. The SALX plates were prepared beforehand separately according to the producer’s manual and used within 5 d. The top cover film of the SALX Plate was opened and the streaking was conducted carefully so as not to break the gel surface. Then the cover film was rolled down to close the SALX Plate carefully not to involve air bubbles and the SALX Plates were incubated at 41.5±1°C for 24±2 h in a horizontal position.

Red/brown colonies with a yellow zone and/or associated gas bubbles were regarded as presumptive *Salmonella* colonies. The SALX Plate was observed from outside and at least 5 presumptive colonies were registered by marking with an ultra-fine tip marker on the top cover film. Then the top cover film was once rolled up and a SALX Disk was inserted between the gel surface and the top cover film. The plate-disk constructs were incubated at 41.5±1°C for 45 h and the color change of the colonies was observed. When red/brown changed to green blue, blue, dark blue, or black, the colony was assigned as *Salmonella* spp.

### 2.6 NIHSJ-01

NIHSJ-01: 2019 developed by the Committee for “The Methods for the Microbiological Examination of Foods”, National Institute of Health Sciences Japan is an alternative to ISO 6579:2002(E). The test protocol of NIHSJ-01 is roughly as follows.

A 25 g test portion was combined with 225 mL buffered peptone water (BPW) for pre-enrichment culture in a stomacher bag. After thorough homogenization for 2 min, the stomacher bag was incubated at 37°C for 20±2 h. Next, the selective enrichment culture was conducted by inoculating 0.1 mL and 1.0 mL of pre-enrichment culture into 10 mL Rappaport-Vassiliadis (RV) medium and 10 mL tetrathionate (TT) medium, respectively, at 42°C for 22±2 h. After the culture, a small aliquot of each culture was streaked onto DHL agar and CHS. DHL produces black colonies that produce hydrogen sulfide, while CHS produces pink to purple colonies regardless of whether they produce hydrogen sulfide. Therefore, those detected by DHL and CHS are sulfide positive strains and those detected only by CHS are sulfide negative strains. These cultures for isolation were conducted at 37°C for 22±2 h.

Three colonies assigned as presumptive *Salmonella* spp. were tested by confirmation culture tests using a triple sugar iron (TSI) agar medium and a lysine indole motility (LIM) medium, respectively, for culture at 37°C for 22±2 h. In the TSI test, if yellowing, blackening, and gas production were observed in the upper layer and bright red coloring of the slope was observed, the TSI test result was decided to be positive. In the LIM medium, if the entire medium turned purple (lysine positive), motility was positive, and the indole reaction was negative (no color change), the LIM test result was decided to be positive. If both tests were positive, the tested colony was decided to be *Salmonella*.

Further confirmation tests were not conducted in this study, since *Salmonella* strains added to the test portions were only two specific strains, FSD284 and FSD347, and moreover, no false positive colony was generated due to non-specific contaminating bacteria.

### 2.7 Estimation of LOD

Theoretical background of LOD was well described elsewhere^6^^,^^7^^)^ and summarized in Annex of ISO 16140: 2016^12^^)^. Fundamentally, the statistical analysis is based on the Poison distribution and final goal of the analysis is the estimation of F value, that is an indicator reflecting typically the effect of food matrix on the sensitivity. LOD_50_ and LOD_95_ are given by the equation:

LOD_50 _= -Ln(1-0.50)/F = 0.69/F, and LOD_95
_= -Ln(1-0.95)/F = 3.00/F.

LOD becomes smaller (more sensitive) as F becomes greater.

In this study, the acceptable performance of a test method was set as the detection of one target cell in a test portion at a higher probability than 95%, namely LOD_95_<1.0 cfu/tp. A downloadable program for LOD_95_ is provided at ISO website^8^^)^. However, all of our data obtained from contaminated test portions were positive and therefore, unable to be analyzed directly. Based on the negative results obtained with blank test portions, we introduced hypothetical results that may be obtained under extremely low concentrations of bacteria. The specific process of analysis was described in detail below in the Annex.

## 3. Results

### 3.1 Determination of the CFP of Single Cell Sorted

**Fig. 2.**
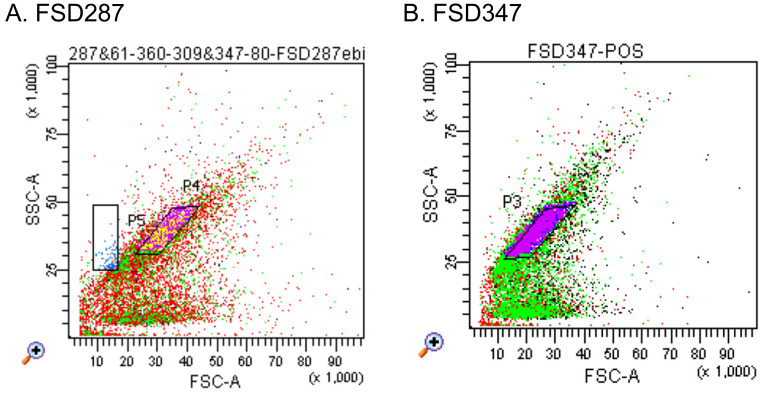
Flow cytograms of Salmonella. FSC: forward scatter, SSC: side scatter. Larger FSC indicates larger cell size and larger SSC suggests higher cell viability. Proper gates determined beforehand for sorting of cells with high CFP: P4 in (A) for FSD287, and P3 in (B) for FSD347.

**Fig. 3.**
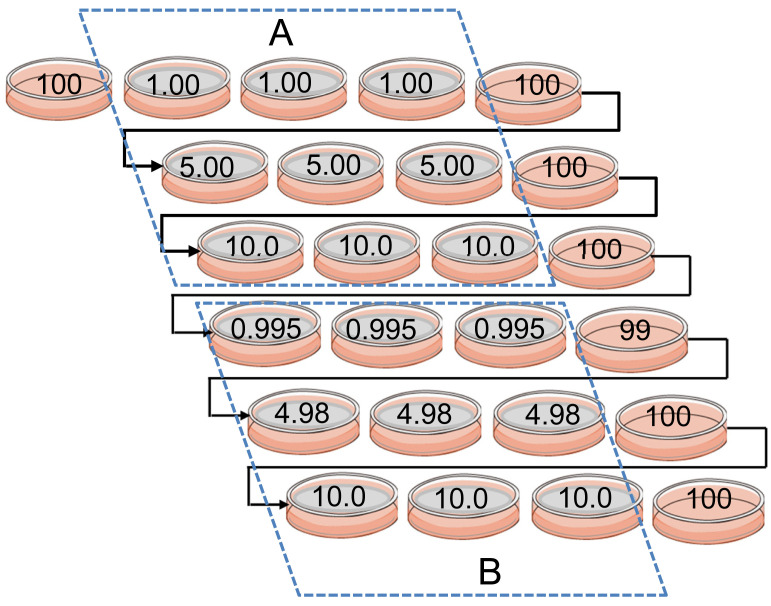
Estimated number of FSD 287 cells with **c**olony forming units in beef test portions. Two sets (A: NIHSJ-01, B: SALX System) of nine-test portions (1 cfu×3, 5 cfu×3, 10 cfu×3) were prepared sequentially. Nine-test portions were prepared for FSD 347 on beef, FSD 287 on shrimp, and FSD 347 on shrimp, respectively in the same manner.

Flow cytograms of FSD287 and FSD347 strains were obtained (**Fig. 2**). The 10×10 cell sorting was conducted on TSA medium. Proper gate conditions were determined as P4 for FSD 287 and P3 for FSD 347, respectively. Under these conditions, cell sorting was conducted sequentially as illustrated in **Fig. 3**. The CFPs were estimated from the results of 10×10 cell sorting before and after the sorting on a three-test portion set (1, 5, 10 cfu/tp) (**Table 1**). The colony forming units in respective test portions were determined as 1×CFP, 5×CFP, and 10×CFP, respectively. CFP was higher than 0.960 for all test portions. Therefore, the real number of cells sorted in test portions were confirmed as 1, 5, and 10 cells, respectively with confidence of higher than 96%.

**Table 1.** CFP determined by 10×10 cell sorting method.

Test method	Strain	*Salmonella* FSD 287		*Salmonella* FSD 347	
	Food	Beef	Shrimp	Beef	Shrimp
NIHSJ-01	tp for 1 cfu	1.000	0.975	0.985	0.995
	tp for 5 cfu	1.000	0.985	0.980	0.980
	tp for 10 cfu	1.000	0.980	0.990	0.960
SALX system	tp for 1 cfu	0.995	0.980	0.990	0.970
	tp for 5 cfu	0.995	0.985	0.970	0.980
	tp for 10 cfu	1.000	0.980	0.960	0.980

### 3.2 Performance of SALX System and NIHSJ-01 to Detect Small Number of *Salmonella* Cells in Foods

The qualitative test results by the two test methods are summarized in **Table 2A** for beef and **Table 2B** for shrimp. For FSD 287, all test portions showed positive results, whereas the blank test portions showed negative results. These demonstrate that both methods can detect a single cell of *Salmonella* in the 25 g test portions both in beef and shrimp. For FSD347, a hydrogen sulfide negative strain, all test portions showed positive results by SALX system and NIHSJ-01 using CHS medium, but not by NIHSJ-01 using DHL medium. It should be noted that such a sensitive detection was specific to *Salmonella*, because contamination of nonspecific viable cells at higher than 10^6^ cfu/25g-tp did not hinder the detection performance. *Salmonella* belongs to the *Enterobacteriaceae* family. If other *Enterobacteriaceae* members than *Salmonella* are present in the contaminating bacteria in the food samples, this may affect the detection of *Salmonella*. In some cases, the growth of *Salmonella* may be inhibited^13^^,^^14^^)^. In this study, *Salmonella* could be detected at a high sensitivity of 1 cfu. Therefore, even if other *Enterobacteriaceae* species were present in the contaminating bacteria, they did not affect the high sensitivity to *Salmonella*.

**Table 2.** Detection rates of small number of *Salmonella* cells in foods

**A.** Beef meat										
Contaminated non-target cells			1.35×10^7^ cfu/25g-tp							
*Salmonella* strain			FSD 287 (H_2_S (+))				FSD 347 (H_2_S (-))			
Number of cells sorted			0	1	5	10	0	1	5	10
Expected number of cfu/25g-tp				1.00	5.00	10.00		1.00	4.98	10.00
Positive/ Inoculated	SALX system		0/3	3/3	3/3	3/3	0/3	3/3	3/3	3/3
	NIHSJ-01	DHL	0/3	3/3	3/3	3/3	0/3	0/3	0/3	0/3
		CHS	0/3	3/3	3/3	3/3	0/3	3/3	3/3	3/3

**Table 2.** 

**B.** Shrimp										
Contaminated non-target cells			3.25×10^6^ cfu/25g-tp							
*Salmonella* strain			FSD 287 (H_2_S (+))				FSD 347 (H_2_S (-))			
Number of cells sorted			0	1	5	10	0	1	5	10
Expected number of cfu/25g-tp				0.98	4,93	9.80		0.98	4.93	9.80
Positive/ Inoculated	SALX system		0/3	3/3	3/3	3/3	0/3	3/3	3/3	3/3
	NIHSJ-01	DHL	0/3	3/3	3/3	3/3	0/3	0/2*	0/3	0/3
		CHS	0/3	3/3	3/3	3/3	0/3	2/2*	3/3	3/3

*1 test portion was lost due to experimental problem.

### 3.3 Single Cfu Detection Ability of SALX System and NIHSJ-01

When FSD 287 was added to beef, the positive rate for three test portions at three different cell concentrations L: 1, M: 5, H: 10 cfu/tp were L: 3/3, M: 3/3, H: 3/3 in both test methods. All nine test portions were positive. The LOD analysis method based on Poisson distribution cannot obtain a definite solution unless there is at least one negative result. Therefore, based on the results that all blank test portions were negative (B: 0/3), we introduced hypothetical data to be obtained under extremely low concentration of target cells. According to the analysis described under Annex, the following results were obtained.

The cell concentration was expressed as A_0_d cfu/tp, where A_0_ is 25 g/tp, the amount of a test portion and d cfu/g is the cell concentration. When A_0_d was changed from 0.1 to 0.0001, LOD_95_ changed from 1.25 cfu/25g-tp to 0.326 cfu/25g-tp (Table 3). Therefore, LOD_95_ for the cases of B:0/3, L:3/3, M:3/3, H:3/3 and B:0/3, L:2/2, M:3/3, H:3/3 was estimated to be lower than 1.0 cfu/25g-tp.

**Table 3.** Changing of LOD_95_ as A_0_d of hypothetical conditions change from 0.1 to 0.0001 cfu/tp

Hypothetical A_0_d [cfu/25g-tp]	F value	LOD_95_ [cfu/25g-tp]
0.1	2.40 (2.04)*	1.25 (1.47)
0.01	4.62 (4.22)	0.648 (0.711)
0.001	6.91 (6.50)	0.434 (0.461)
0.0001	9.20 (8.85)	0.326 (0.339)
0	>9.20 (>8.85)	<0.326 (<0.339)

*Numbers in parentheses are results for the case of Shrimp/NIHSJ-01/CHS/FSD347 in Table 2B. No result was obtained with case of Shrimp/NIHSJ-01/DHL/FSD347 in Table 2B. Numbers outside parentheses are results for the other cases in Table 2A and 2B.

Regarding selectivity for bacterial species, NIHSJ-01 can selectively detect hydrogen sulfide-producing bacteria by using DHL as a selective isolation medium.

## 4. Discussion

The European and International Standard method for the detection of *Salmonella* spp. in samples from the primary production stage (EN ISO 6579:2002) was revised in 2017. The performance characteristics of EN ISO 6579-1:2017^15^^)^ were determined based not only on the results of the interlaboratory study carried out in 2013, but also on several other interlaboratory studies. These performance characteristics consist of specificity, sensitivity and LOD_50_, that were calculated by Mooijman et al.^16^^)^. LOD_50_ for fresh cheese curd, egg powder, and raw poultry meat were 5.7, 6.0, and 2.2 (cfu/25g-tp), respectively. On the other hand, in this study, the LOD_50_ values for beef and shrimp were much lower (<0.075 cfu/25g-tp). This is thought to be because the test portions did not contain any contaminants such as *Enterobacteriaceae* that could affect the detection of *Salmonella*. Therefore, the performance of the test method can be evaluated significantly higher by introducing an evaluation method using a highly accurate viable bacterium standard material, which may open the way of application of the test method to critical cases requiring high sensitivity.

The accuracy of viable bacterial standard material depends upon the performance of cell sorter and associate devices. Over the past decade, the performance of cell sorters has been improved significantly. In the past, the focus was on large animal cells, but recently, cell sorters from several manufacturers have become capable of easy sorting of bacteria at single cell level. Such a technological progress appears to be due to the growing need for bacterial sorting.

Recently, for instance, there is a need of a rapid non-culture assay system for identifying rare species and slow-growing taxa. Instead of conventional flasks and multi-well plates, the idea of an alternative, ultra-high-throughput droplet microfluidic screening assay was proposed^17^^)^. It is also required to rapidly classify and identify antibacterial-resistant bacteria. In order to meet this requirement, a combined system of a cell sorter for bacterial detection with an elastic light scattering method for bacterial classification, and further with machine learning methods was proposed^18^^)^. At the same time, with the need to handle such a wide variety of bacteria, cleaning and sterilization of the cell sorter has become an important issue. It was sought to better understand how cell sorters were maintained and evaluated for contaminants such as bacteria, endotoxin, and RNases. In addition, the efficacy of an endotoxin decontamination method was evaluated^19^^)^.

Considering these trends, the development of bacterial cell sorting technologies will be accelerated by the needs that require them, and vice versa. The present study suggests the importance of the rapid preparation system for standard material of single bacterial cell with different levels of viability, from full viable to injured state^20^^)^. Such a need also may contribute to the acceleration of technological innovation in single-cell sorting systems.

## Annex

When the bacterial concentration is low, the spatial distribution of bacteria can be assumed to follow a Poisson distribution, and the relationship between concentration (d [cfu/g]) and detection probability (p) is expressed as

p =
1-exp(-FA_0_d) (1)

where A_0_ is the quantity of a test portion. For example, if A_0_=25 [g], as in this study, the bacterial concentration can be expressed as A_0_d [cfu/tp]. F is a coefficient that represents the effects of foods and various other factors that influence the detection rate. In this study, the control (F=1) means test portions containing no food. If the detection rate is lower than the control, F is smaller than 1.0. Since LOD_95_ is A_0_d at p=0.95, equation (1) becomes

0.95 = 1-exp(-FA_0_d) (2)

This equation may be converted as follows

A_0_d = -Ln(1-0.95)/F =
3.00/F (3)

Assuming that the number of positive test portions is y_j_ when n_j_ repeat test portions are tested at different bacterial concentrations (A_0_d_j_, j=1 to 4), F value is determined to be a value that satisfies the following equation (4).

$$\overset{4}{\underset{\text{j}=1}{\sum }}\left[\frac{{\text{y}}_{\text{j}}{\text{d}}_{\text{j}}}{\exp\left({\text{FA}}_{0}{\text{d}}_{\text{j}}\right)-1}-\left({\text{n}}_{\text{j}}-{\text{y}}_{\text{j}}\right){\text{d}}_{\text{j}}\right]=0\;$$ (4)

Test conditions and results in Table 4 were substituted into this equation (4).

**Table 4.** Number of positive results obtained at different cell concentrations

Cell conc. symbol	Cell conc. number j	Cell conc. (dj) cfu/25g-tp	Number of repeat test portions (n_j_)	Number of positive results (y_j_)
H	1	1	3	3
M	2	5	3	3
L	3	10	3	3
B	4	0	3	0

In usual cases, only the results of H(d_1_=10 cfu/25g-tp), M(d_2_=5 cfu/25g-tp), and L(d_3_=1 cfu/25g-tp) are used in the analysis and the results of B(d_4_=0 cfu/25g-tp) is not included. However, when all the samples of H, M, and L are positive (y_j_=3 at d_j_ for j=1~3), as in this case, F cannot be obtained from (4). Therefore, instead of blank test portions (y_j_=0 at d_4_=0), we introduced hypothetical test portions in which cell concentration (d_4_ cfu/25g-tp) was 0.1 or smaller. When d_4_ was 0.1, for instance, the left side of the equation (4) becomes the following equation by substituting n_j_=y_j_=3 for j=1~3, n_4_=3, y_4_=0, d_1_=10/25 cfu/g, d_2_=5/25 cfu/g, d_3_=10/25 cfu/g, and d_4_=0.1/25 cfu/g.

Leftsideof 4=1025×3exp10×F−1−3−3×1025+525×3exp5×F−1−3−3×525+125×3exp1×F−1−3−3×125+0.125×0exp0.1×F−1−3−0×0.125

**Fig. 4.**
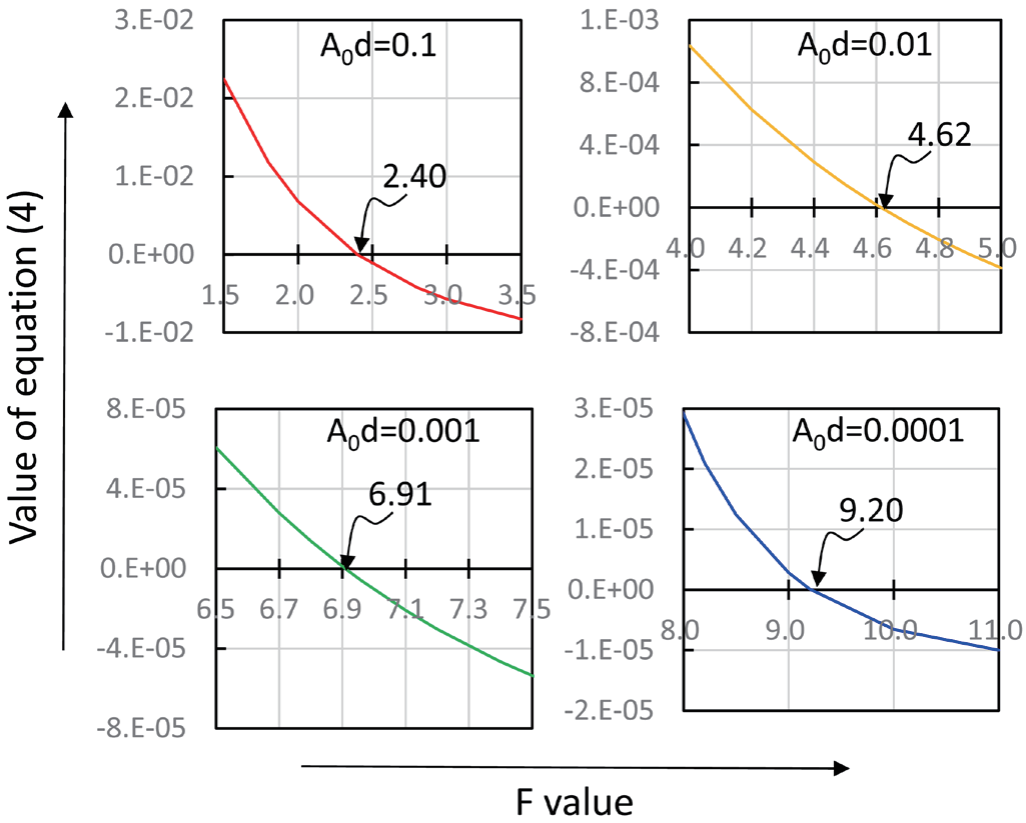
F values determined for various values of A_0_d. A_0_d was varied from 0.1 to 0.0001 to approach zero. In panel for A_0_d=0.1, for instance, F value was varied from 1.5 to 3.5 and the value of the left side of equation (4) was plotted against F values. The F value for A_0_d=0.1 was determined as 2.40, the point where the correlation curve crossed zero.

Next, we plotted the values of the left side of (4) against F. The result is shown in Fig. 4 for A_0_d=0.1 (upper left panel). From this result, the value of F for which the left side of (4) became 0 was 2.40. Similarly, we determined the values of F for A_0_d=0.01, 0.001, and 0.0001 (Fig. 4). The F values listed in Table 4 in the Results section summarize these results.

**Fig. 5.**
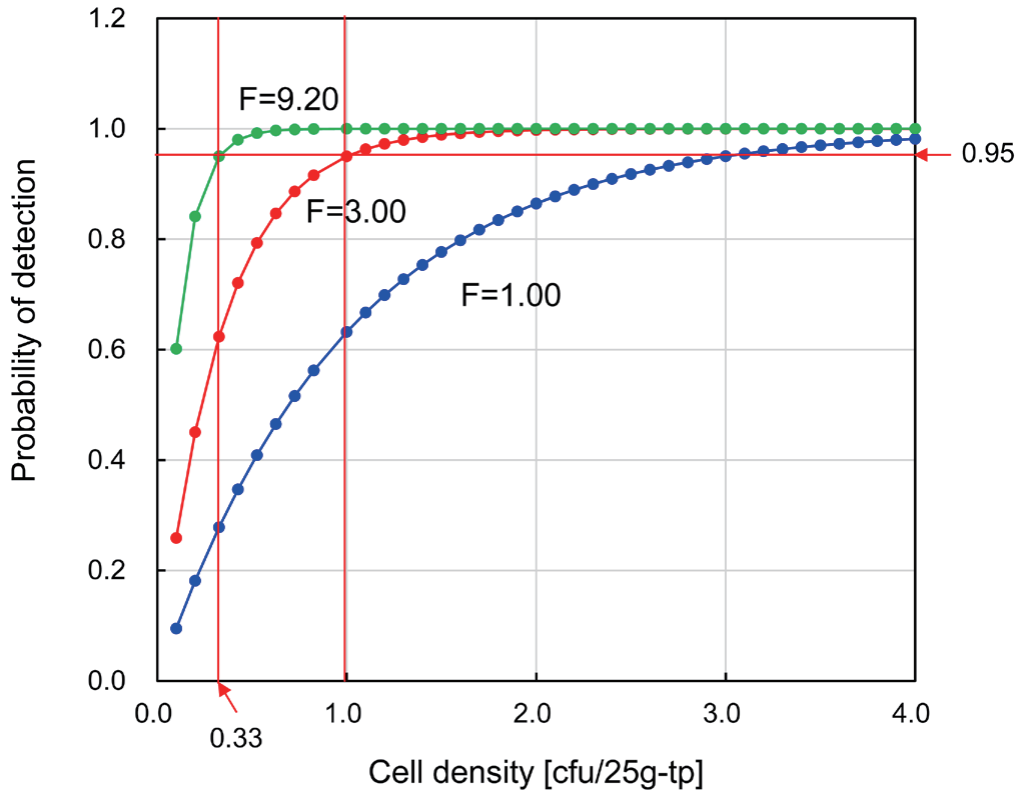
Dependence of F values on the 95% detection probability. X axis: A_0_d cfu/25g-tp, Y axis: p of equation (1). Three curves are plotted for F=1.00 (●), F=3.00 (●), F=9.20 (●). When F=1.00, p at A_0_d=1 cfu/tp was 0.63, indicating LOD_63_=1 cfu/tp. When F=3.00, p at A_0_d=1 cfu/tp was 0.95, indicating LOD_95_=1 cfu/tp. When F=9.20 (hypothetical blank=0.0001 cfu/tp), p at 0.33 cfu/tp was 0.95, indicating LOD_95_=0.33 cfu/tp. When hypothetical blank<0.0001 cfu/tp, F>9.20 and therefore, LOD_95_<0.33 cfu/tp. Consequently LOD_95_<1.00 cfu/tp when blank=0.

From these results, the bacterial detection rates for F=1 (control), F=3.00 (F value for LOD_95_=1.00 cfu/25g-tp), and F=9.20 (F value for A_0_d=0.0001 cfu/25g-tp) are shown in Fig. 5. LOD_95_ is the bacterial concentration at the point where the POD curve intersects with the horizontal line for POD=0.95. As F increases, LOD_95_ decreases. If this value is smaller than 1, it can be concluded that there is a 95% or higher probability of detecting one bacterium in the test portion. From these results, LOD_95_ for the case of B:0/3, L3:3/3, M:3/3, H:3/3 and also for the case of B:0/3, L:2/2, M:3/3, H:3/3 was estimated as <0.326 cfu/tp, and therefore sufficiently <1.0 cfu/tp.

When comparing the performance of qualitative test methods, LOD_50_ is typically compared. Therefore, based on the values in Table 3, we calculated the LOD_50_ for the test methods investigated in this study, which is 0.693/F. That was LOD_50_ <0.075 cfu/25g-tp.

## References

[1] Morgan CA, Bigeni P, Herman N, Gauci M, White PA, Vesey G. Production of precise microbiology standards using flow cytometry and freeze drying. Cytometry A. 2004; 62A(2): 162–168. . 10.1002/cyto.a.20075 15517560

[2] Wohlsen T, Bates J, Vesey G, Robinson WA, Katouli M. Evaluation of the methods for enumerating coliform bacteria from water samples using precise reference standards. Lett Appl Microbiol. 2006; 42(4): 350–356. . 10.1111/j.1472-765X.2006.01854.x 16599987

[3] ISO 16140-3:2021. Microbiology of the food chain — Method validation. Part 3: Protocol for the verification of reference methods and validated alternative methods in a single laboratory. https://www.iso.org/standard/66324.html. Published 2021. Accessed April 1, 2022.

[4] Matsuoka H, Shigetomi T, Funabashi H, Saito M, Igimi S. Tryptic soy medium is feasible for the in situ preparation of standards containing small defined numbers of microbial cells. J Microbiol Methods. 2013; 93(1): 49–51. . 10.1016/j.mimet.2013.01.021 23403310

[5] Matsuoka H, Nakano K, Takatani N, Yoshida T, Igimi S, Saito M. Flow cytometric method for in situ preparation of standard materials of a small defined number of microbial cells with colony-forming potentiality. J AOAC Int. 2014; 97(2): 479–483. . 10.5740/jaoacint.13-302 24830159

[6] Wilrich C, Wilrich PT. Estimation of the POD function and the LOD of a qualitative microbiological measurement method. J AOAC Int. 2009; 92(6): 1763–1772. . 10.1093/jaoac/92.6.1763 20166595

[7] Mărgăritescu I, Wilrich PT. Determination of the relative level of detection of a Qualitative microbiological measurement method with respect to a reference measurement method. J AOAC Int. 2013; 96(5): 1086–1091. . 10.5740/jaoacint.12-377 24282952

[8] ISO website. https://standards.iso.org/iso/16140/-4/ed-1/en/amd/1/PODLOD-ver12.xlsm. Accessed April 1, 2022.

[r9] 9.3M™ Petrifilm™ *Salmonella* Express System. *Official Methods of Analysis of AOAC INTERNATIONAL*. 22nd ed. Latimer GW Jr (ed.). Oxford University Press; 2023. AOAC Official Method 2014.01. https://academic.oup.com/aoac-publications/book/45491?searchresult=1 Accessed April 1, 2020.

[10] Bird P, Flannery J, Crowley E, et al. Evaluation of the 3M™ Petrifilm™ *Salmonella* express system for the detection of *Salmonella* species in selected foods: collaborative study. *J AOAC Int*. 2014; **97(**6): 1563–1575. 10.5740/jaoacint.14-120 25632434

[11] National Institute of Health Sciences. Standard test method for *Salmonella* spp. NIHSJ-01 [in Japanese]. https://www.nihs.go.jp/fhm/mmef/pdf/protocol/NIHSJ-01_2019.pdf. Accessed April 1, 2020

[12] ISO 16140-2:2016. Microbiology of the food chain — Method validation. Part 2: Protocol for the validation of alternative (proprietary) methods against a reference method. https:// www.iso.org/standard/54870.html. Published 2016. Accessed April 1, 2020.

[13] Velazquez EM, Nguyen H, Heasley KT, et al. Endogenous *Enterobacteriaceae* underlie variation in susceptibility to *Salmonella* infection. Nat Microbiol. 2019; 4(6): 1057–1064. . 10.1038/s41564-019-0407-8 30911125 PMC6533147

[14] Litvak Y, Mon KKZ, Nguyen H, et al. Commensal *Enterobacteriaceae* protect against *Salmonella* colonization through oxygen competition. Cell Host Microbe. 2019; 25(1): 128–139.e5. . 10.1016/j.chom.2018.12.003 30629913 PMC12036633

[15] ISO 6579-1:2017. Microbiology of the food chain — Horizontal method for the detection, enumeration and serotyping of Salmonella. Part 1: Detection of Salmonella spp. https:// www.iso.org/standard/56712.html. Published 2017. Accessed April 1, 2020 10.1016/j.ijfoodmicro.2018.03.022 29803313

[16] Mooijman KA, Pielaat A, Kuijpers AFA. Validation of EN ISO 6579-1 - Microbiology of the food chain - Horizontal method for the detection, enumeration and serotyping of *Salmonella* - Part 1 detection of *Salmonella* spp. Int J Food Microbiol. 2019; 288: 3–12. . 10.1016/j.ijfoodmicro.2018.03.022 29803313

[17] Potenza L, Kozon L, Drewniak L, Kaminski TS. Passive droplet microfluidic platform for high-throughput screening of microbial proteolytic activity. Anal Chem. 2024; 96(40): 15931–15940. . 10.1021/acs.analchem.4c02979 39320273 PMC11465220

[18] Narayana Iyengar S, Dowden B, Ragheb K, et al. Identifying antibiotic-resistant strains via cell sorting and elastic-light-scatter phenotyping. Appl Microbiol Biotechnol. 2024; 108(1): 406. . 10.1007/s00253-024-13232-0 38958764 PMC11222266

[19] Box A, Holmes L, DeLay M, et al. Cell sorter cleaning practices and their impact on instrument sterility. *J Biomol Tech*. 2022; **33**(1):3fc1f5fe.e2675d74. 10.7171/3fc1f5fe.e2675d74 PMC9258916 35837003

[20] Saito M, Takatani N, Yoshida T, Mariogani A, Cho E, Matsuoka H. Effects of injured and dead cells of *Escherichia coli* on the colony‐forming rate of live cells. FEBS Open Bio. 2021; 11(2): 404–412. . 10.1002/2211-5463.13051 33264499 PMC7876490

